# Small-scale protocol for magnetic coagulation testing

**DOI:** 10.1016/j.mex.2025.103550

**Published:** 2025-08-05

**Authors:** Hans Kristianto, Wibawa Hendra Saputera, Johnner P. Sitompul

**Affiliations:** a Doctoral Program of Chemical Engineering, Faculty of Industrial Technology, Institut Teknologi Bandung, Jl. Ganesha no 10, Bandung, 40132, Indonesia; b Department of Chemical Engineering, Faculty of Engineering Technology, Parahyangan Catholic University, Jl. Ciumbuleuit 94, Bandung 40141, Indonesia; c Department of Chemical Engineering, Faculty of Industrial Technology, Institut Teknologi Bandung, Jl. Ganesha no 10, Bandung, 40132, Indonesia; d Research Center for New and Renewable Energy, Institut Teknologi Bandung, Jl. Ganesha no 10, Bandung, 40132, Indonesia; e Center for Catalysis and Reaction Engineering, Institut Teknologi Bandung, Jl. Ganesha no 10, Bandung, 40132, Indonesia

**Keywords:** Coagulation, Congo red, Jar test, Magnetic coagulant

## Abstract

Coagulation is a key process in water and wastewater treatment, typically evaluated using a standard jar test. However, this method requires large volumes of reagents and extended testing time, making it impractical in certain conditions. This study presents a novel miniaturized coagulation method using only 5 mL of wastewater to assess the performance of a magnetic coagulant synthesized from *Moringa oleifera* seed extract and Fe_3_O_4_ nanoparticles. Congo red dye served as the model pollutant, and the removal efficiency of the mini test was compared with the conventional jar test under identical conditions. The miniaturized method achieved a removal efficiency of 94.23 ± 0.611 %, which is slightly lower than the 97.06 ± 0.746 %, achieved by the conventional method. While statistical analysis indicated a significant difference (p < 0.05), the absolute difference (∼2.83 %) was minor and did not affect practical performance.

• The proposed method offers significant advantages, including reduced reagent consumption, a lower sample volume, minimized waste generation, and enabled rapid screening of coagulant performance.

• This approach aligns with green chemistry and engineering principles and serves as an efficient tool for early-stage coagulant screening, particularly in low-resource settings as well as high-throughput testing environments.

## Graphical abstract

**Figure:**
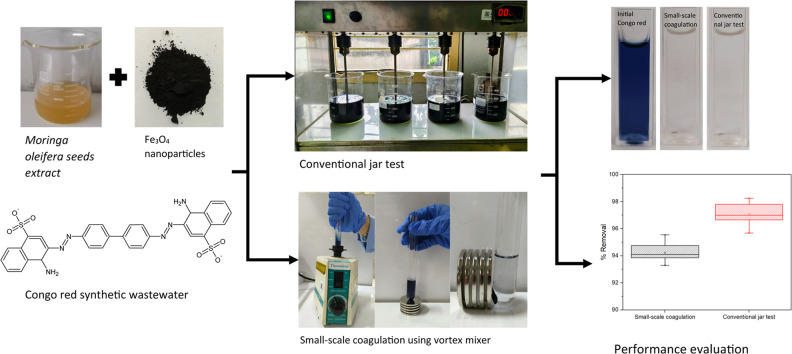

**Specifications table**

**Table:** 

**Subject area**	Chemical Engineering
**More specific subject area**	Coagulation and flocculation
**Name of your method**	Small-scale coagulation test method
**Name and reference of original method**	Ghebremichael, K. A., Gunaratna, K. R., Henriksson, H., Brumer, H., & Dalhammar, G. (2005). A simple purification and activity assay of the coagulant protein from Moringa oleifera seed. *Wat Res, 39*, 2338–2344. https://doi.org/10.1016/j.watres.2005.04.012
**Resource availability**	Data will be made available upon request to the corresponding author.

## Background

Access to clean water remains a global priority, with coagulation playing a vital role in water and wastewater treatment by removing suspended solids, turbidity, organic matter, and other contaminants. Inorganic coagulants such as aluminum and iron salts are commonly employed to destabilize colloids promoting floc formation and subsequent sedimentation. While this process is effective and widely used, reliance on inorganic coagulants presents several drawbacks such as production of non-biodegradable sludge and occurrence of residual metal ions in treated water, which have been associated with potential health risks (e.g. Alzheimer’s disease, dementia) [1]. To address these drawbacks, increasing attention has been given to natural coagulant derived from plant, animal, and microbial sources [2]. Among these, the *Moringa oleifera* seed extract has emerged as a promising alternative. Furthermore, the integration of natural coagulants with magnetic iron oxide nanoparticles is gaining interest for enhancing coagulation efficiency by producing larger flocs with faster settling times [3,4].

In laboratory settings, coagulation parameters such as pH and coagulant dosage are typically evaluated using a standard jar test method (ASTM D2035-19), which involves agitating multiple 0.5–1 L samples in parallel beakers. Although effective in simulating full-scale mixing conditions, the jar test is resource-intensive, requiring large volumes of samples and coagulants. This makes it time-consuming and inefficient, especially when screening a wide range of experimental conditions. A down-scaled coagulation method could address these challenges by reducing reagent and sample consumption while accelerating testing, offering a rapid and reliable alternative to the standard jar tests protocols.

Several studies have explored small volume coagulation approaches. Ghebremichael, Gunaratna, Henriksson, Brumer and Dalhammar [5] utilized a 1 mL clay suspension in a cuvette, requiring only 10 μL *Moringa oleifera* extract as a coagulant, effectively replacing the standard jar test. This method was subsequently adopted in other studies [6,7], though without detailed protocols or validation data to support its broader application. Aboagye, Navele and Essuman [8] conducted coagulation tests using 150 mL river water samples with *Moringa oleifera* seeds powder, while a more recent study, namely Oliveira, Nascimento and Donadel [9] reported coagulation tests using *Moringa oleifera*-based natural coagulants in water volumes ranging from 40 to 65 mL for clay suspensions.

Here we present a novel miniaturized coagulation testing method using only 5 mL samples is presented, using Congo red synthetic wastewater as the dye model. The magnetic coagulant is synthesized from a combination of *Moringa oleifera* seeds extract and Fe_3_O_4_ nanoparticles. Designed for rapid and resource-efficient screening, this method offers high replicability and produces results comparable to the standard jar test method. Furthermore, the use of small sample volume simplifies the procedure, minimizes reagent consumption, and significantly reduces waste generation, providing a more sustainable method to coagulation testing.

## Method details

**1. Preparation of Congo red synthetic wastewater**

The Congo red synthetic wastewater was prepared using the following procedure:

1. Weigh 1 g of Congo red powder (Sigma-Aldrich) using an analytical balance.
2. Dissolve the powder in distilled water to prepare a 1 g/L stock solution.
3. Pipette 20 mL of the stock solution and dilute to 1 L with distilled water to obtain a 50 mg/L working solution.
4. Adjust the pH of the working solution to the desired value using 0.1 N HCl or 0.1 N NaOH prior to use.

**2. Preparation of *Moringa oleifera* crude extract**

The crude extract *Moringa oleifera* was prepared as follows:

1. Weigh 5 g of dried *Moringa oleifera* seed kernel powder.
2. Add the powder to 100 mL of 1 M NaCl solution.
3. Stir the suspension using magnetic stirrer at 100 rpm for 30 min.
4. Centrifuge the mixture at 6000 rpm for 10 min to separate solids.
5. Filter the supernatant to remove remaining particulates.
6. Use the resulting extract directly as a natural coagulant
7. Prepare fresh extract prior to each experiment to maintain the activity of the coagulant agents.

**3. Preparation of magnetic coagulant**

Magnetic coagulant was prepared by dispersing iron oxide nanoparticles (IONPs) into the *Moringa extract seed*. A concentration of 3 mg/mL extract of Fe_3_O_4_ nanopowder (Aldrich) was employed [10].

1. Measure 20 mL of *Moringa* seed extract.
2. Add the required amount of IONPs to the extract to achieve the desired concentration. In this study 60 mg Fe_3_O_4_ nanopowder was used.
3. Sonicate the mixture in an ultrasonic bath (Elmasonic S300H Sonicator) for 5 min to ensure proper dispersion of IONPs.
4. Use the resulting suspension as a magnetic coagulant in subsequent experiments.

**4. Coagulation study using jar test**

A conventional jar test experiment was conducted to serve as a benchmark for the miniaturized test. The conditions used were: pH 3 and coagulant dose of 20 mL/L wastewater [10].

1. Prepare 500 mL of 50 mg/L Congo red solution in a 1 L beaker.
2. Adjust the solution pH to 3 using 0.1 M HCl or 0.1 M NaOH, prior to coagulation.
3. Add the magnetic coagulant in the required dosage.
4. Conduct coagulation/flocculation: rapid mixing at 200 rpm for 3 min, followed by slow mixing at 60 rpm for 30 min to promote floc formation.
5. Allow the mixture to settle for 1 h with an external magnetic field using neodymium magnets placed beneath the beaker.
6. Collect the supernatant from 2 to 3 cm below the surface for further analysis.

**5. Small-scale coagulation study**

The miniaturized coagulation study was carried out in test tubes using only 5 mL of synthetic wastewater sample. Conditions matched those of the jar test (pH 3, 20 mL/L coagulant dose), requiring only 100 μL of magnetic coagulant for one batch of test. The detailed procedure is as follow:

1. Pour 5 mL of Congo red wastewater (pH 3) into a test tube.
2. Pipette 100 µL of the magnetic coagulant using a micropipette.
3. Add the coagulant to the test tube and mix using a vortex mixer (Thermolyne type 37,600 mixer) for 10 s to ensure proper dispersion.
4. Allow the mixture to settle for 10 min with a neodymium magnet (diameter 40 mm, height 3 mm, magnetic flux density 65 mT) placed beside the test tube.
5. Carefully withdraw 3 mL of the supernatant for analytical measurement.

**Fig. 1:**
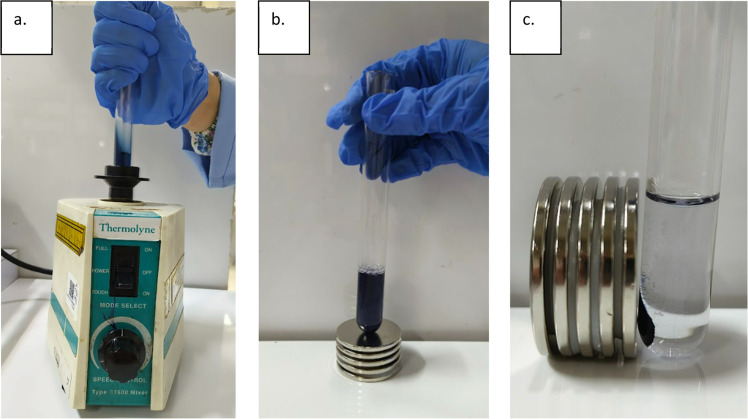
Small-scale coagulation process: mixing with vortex mixer (a), settling after coagulation (b), and magnetic floc separation using neodymium magnets (c).

Illustration of this method is presented in Fig. 1.

**6. Evaluation of the coagulation performance**

The coagulation performance for each method was assessed based on the percentage removal of the Congo red. The procedure is as follows:

1. Measure the initial concentration (Ci, in mg/L) of Congo red solution before coagulation using a visible spectrophotometer (Thermo Scientific Genesys 150)
2. Set the detection wavelength to 575 nm (maximum absorbance for Congo red).
3. Use distilled water as the blank for calibration.
4. After coagulation and settling, measure the final concentration (Cf, in mg/L) under the same conditions.
5. Calculate the percentage removal using Eq. (1):
  (1) $$\%\;removal=\;\frac{Ci-Cf}{Ci}\;\times \;100\%$$

**7. Visual observation of formed flocs using an optical microscope**

Optical microscopy was employed to visually confirm floc formation after coagulation and settling. The procedure is as follows:

1. Immediately after settling, collect a drop pf the sample using a micropipette to preserve floc structure.
2. Place the drop on a clean glass microscope slide.
3. Gently cover with a coverslip, avoiding air bubbles and applying minimal pressure to maintain floc integrity.
4. Observe under an optical microscope (Rehaze Microscope Binocular XSZ-107BN) at 400 × total magnification
5. Adjust focus and illumination to clearly observe floc size, shape, and distribution.
6. Capture images using a camera-mounted microscope for documentation.
7. Analyze the 2-dimension (2-D) fractal dimension (D_f_) of the flocs by using ImageJ software (version 1.54 g) with FracLac plugin (version 2.5).

## Method validation

**Fig. 2:**
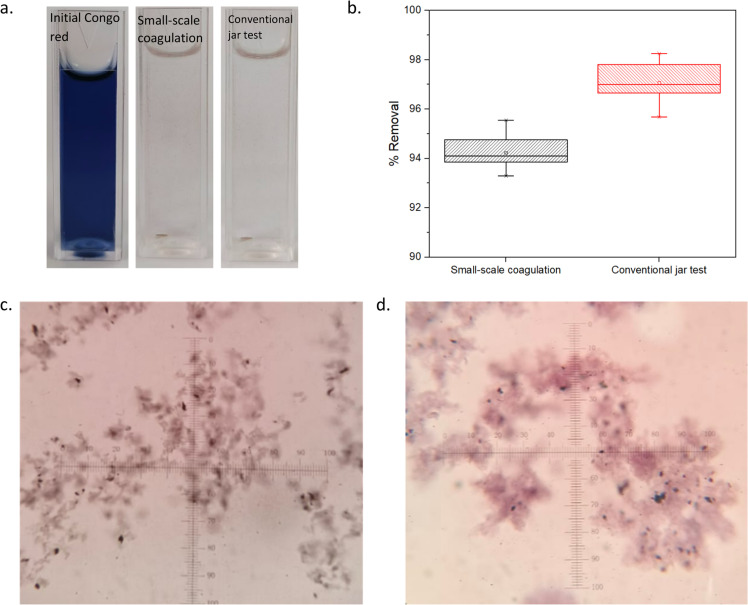
Visual observation of samples before and after coagulation treatment (a), the box-plot of Congo red removal employing the small scale and conventional jar test methods (b), and optical microscopy images of flocs formed in the small-scale method (c) and jar test (d) at 400 × magnification.

The small-scale coagulation method was validated by comparing results from 15 independent batches with the conventional jar method. Validation involved visual comparison treated solution, Congo red removal efficiency, and microscopic analysis of flocs, as shown in Fig. 2.

Fig. 2.a illustrates the visual comparison of Congo red solution before and after coagulation treatment using both small-scale and conventional jar test methods. The untreated solution appeared deep blue, indicative of high Congo red dye concentration at pH 3. Post treatment, both the small-scale and conventional jar test methods produced significantly clearer solutions with minimal residual color or suspended solids, demonstrating effective dye removal. Fig. 2.b quantifies the removal efficiency. From the box-plot, it can be observed that the small-scale coagulation presented a narrower interquartile range, compared to the jar-test results that showed less variability of the data. This is consistent with its smaller standard deviation. Furthermore, both data showed relatively symmetrical distributions, although a slight skewness was observed in the jar-test results. The small-scale method achieved a mean removal of 94.23 ± 0.611 %, while the conventional jar test showed a slightly higher removal of 97.06 ± 0.746 %.

Fig. 2.c-d shows microscopic observation of flocs formed in both methods. In both cases, flocs were visible and exhibited fractal-like morphologies. Fine black particles, attributed to Fe_3_O_4_ nanoparticles, were observed within the flocs, confirming successful integration of the magnetic component. However, morphological differences were noted: flocs from the small-scale test were finer, more dispersed, and less compact, whereas those from the conventional jar test were larger, denser, and more aggregated. These differences are likely attributed to the variations in hydrodynamic conditions. The conventional method benefits from sequential rapid and slow mixing, followed by extended settling time, which facilitates larger and denser floc formation. In contrast, the small-scale system operates under milder mixing and shorter settling time, which limits floc growth and compaction [11].

**Fig. 3:**
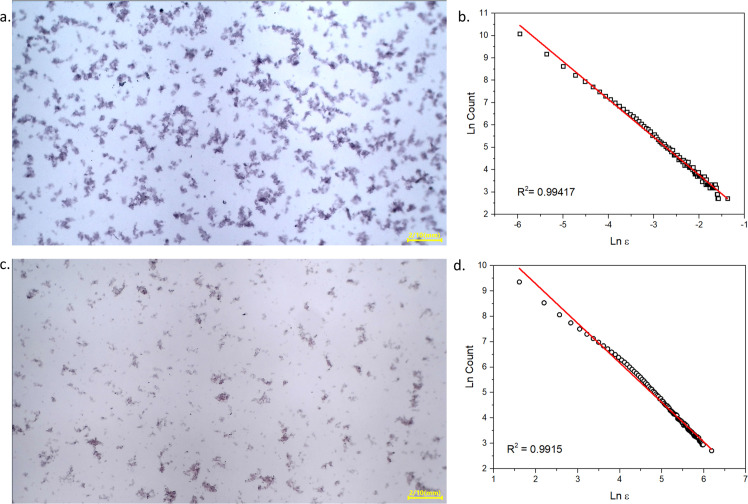
Optical microscopy images of flocs formed in the jar test (a) and small-scale method (c) and its corresponding relationship of floc count and scale - ɛ (b; d).

To quantify the differences between the floc formation, a two-dimensional (2-D) fractal dimension (Df) analysis was applied to the corresponding optical microscopy images, as presented in Fig. 3. The floc images were analyzed using the FracLac plugin in ImageJ using the box-counting method, which is a widely used approach for estimating the 2-D fractal dimension [12]. The analysis revealed that the conventional jar-test flocs’ average Df value amounted to 1.7031, whereas the small-scale coagulation produced flocs with a lower average Df value of 1.5613. The 2-D fractal dimension typically ranges from 1.0 to 2.0, with higher values indicating more compact and dense floc structures. In general, the obtained Df values in this study are comparable to Df values reported in previous studies, where they commonly ranged from 1.30–1.90 [[13], [14], [15]]. The lower Df value observed in the small-scale coagulation further suggests a less compact floc structure, corroborating the visual differences noted in Fig. 2.c–d.

Statistical analyses were made using SPSS Statistics (version 20). Normality of the data was assessed by using the one-sample Kolmogorov Smirnov test, as shown in Table 1. Both the small-scale coagulation and conventional jar test removal data sets were found to be normally distributed, indicated by p-values greater than 0.05 This result justified the application of a paired *t*-test to compare the two methods. To evaluate the consistency between the small-scale coagulation method and the conventional jar test, a paired *t*-test was conducted on the removal efficiency data, as summarized in Table 2. The analysis revealed a statistically significant difference between the two methods, with a calculated t-statistic of –13.614, which exceeds the t-critical value. The two-tailed p-value was 0.000 (<0.05), which is well below the significance threshold of 0.05 at a 95 % confidence level. These results indicate that the mean removal efficiency of the small-scale method (94.23 %) is statistically different from that of the conventional jar test (97.06 %).

**Table 1:** One-sample Kolmogorov-Smirnov test.

**Parameters**		**Small-scale coagulation**	**Conventional jar test**
N		15	15
Normal Parameters^a^^,^^b^	Mean	94.2334	97.0699
	Std. Deviation	.61181	.74623
Most Extreme Differences	Absolute	.175	.170
	Positive	.175	.170
	Negative	−0.082	−0.104
Kolmogorov-Smirnov Z		.677	.657
Asymp. Sig. (2-tailed)		.749	.781

a . Test distribution is normal.

b . Calculated from data.

**Table 2:** Summary of paired *t*-test results comparing removal efficiencies between the small-scale coagulation method and the conventional jar test.

		Paired Differences					t	df	Sig. (2-tailed)
		Mean	Std. Deviation	Std. Error Mean	95 % Confidence Interval of the Difference				
					Lower	Upper			
Pair 1	Small-scale coagulation − Conventional jar test	−2.83651	.80693	.20835	−3.28337	−2.38965	−13.614	14	.000

Although the difference is statistically significant, the absolute difference of 2.83 % in removal efficiency is relatively minor in practical terms, especially when weighed against the benefits offered by the small-scale approach. This highlights the distinction between statistical and practical significance: while the *t*-test confirms a significant difference at the 95 % confidence level (*p* < 0.05), the magnitude of this difference is small enough to be considered negligible in many real-world scenarios. In practice, such a marginal efficiency reduction could be acceptable when balanced against advantages such as time savings, reduced sample and reagent volumes, and faster throughput. The complete coagulation test using the small-scale method requires only 10 min, compared to over 90 min for the conventional jar test. Additional benefits include significantly reduced reagent and sample volume requirements, faster screening capability, and minimal waste generation. These findings suggest that while the small-scale method may not fully replace the conventional jar test in all applications, it offers sufficiently comparable performance to serve as a reliable and efficient tool for preliminary coagulation screening, particularly when rapid results and resource efficiency are prioritized.

## Conclusions

In this study, we demonstrate the application of a small-scale coagulation testing method as an alternative to the conventional jar test, using Congo red synthetic wastewater as the model pollutant, and a magnetic coagulant composed of *Moringa oleifera* seeds extract and Fe_3_O_4_ nanoparticles. The small-scale method achieved an average removal efficiency of 94.23 %, which is slightly lower than the 97.06 % obtained using the standard jar test. A paired *t*-test confirmed a statistically significant difference between the two methods, however, the absolute difference of 2.83 % is minor in practical applications. The comparable removal performance and visual clarity suggest that the small-scale approach is both feasible and reliable for rapid screening and optimization for coagulation processes. This miniaturized method offers notable benefits, including reduced reagent consumption, minimal waste generation, and higher throughput potential, aligning well with green chemistry and engineering principles. It is particularly well-suited for exploratory research, early-stage testing, and resource limited conditions.

## Limitations

While the miniaturized coagulation method demonstrated promising results, this study remains subject to several limitations. First, the experiments were conducted under a single set of operating conditions, i.e. a fixed pH and coagulant dose. Additionally, the study utilized synthetic wastewater containing Congo red as the model pollutant, which may not fully represent the complexity of real wastewater. Evaluation of various wastewater types and a boarder range of coagulation conditions will be the focus of future research.

## Ethics statements

Not applicable.

## Supplementary material *and/or* additional information [OPTIONAL]

None.

## CRediT authorship contribution statement

**Hans Kristianto:** Conceptualization, Formal analysis, Methodology, Visualization, Writing – original draft. **Wibawa Hendra Saputera:** Conceptualization, Supervision, Writing – review & editing. **Johnner P. Sitompul:** Conceptualization, Supervision, Writing – review & editing.

## Declaration of competing interest

The authors declare that they have no known competing financial interests or personal relationships that could have appeared to influence the work reported in this paper.

## Data Availability

Data will be made available on request.
